# Novel alleles in the era of next-generation sequencing-based HLA typing calls for standardization and policy

**DOI:** 10.3389/fgene.2023.1282834

**Published:** 2023-10-13

**Authors:** Jenny N. Tran, Karen R. Sherwood, Ahmed Mostafa, Rey Vincent Benedicto, Allaa ElaAlim, Anna Greenshields, Paul Keown, Robert Liwski, James H. Lan

**Affiliations:** ^1^ British Columbia Provincial Immunology Laboratory, Vancouver Coastal Health, Vancouver, BC, Canada; ^2^ Department of Pathology and Laboratory Medicine, University of Saskatchewan, Saskatoon, SK, Canada; ^3^ Department of Pathology, Dalhousie University, Halifax, NS, Canada; ^4^ Department of Pathology and Laboratory Medicine, Vancouver Coastal Health, University of British Columbia, Vancouver, BC, Canada

**Keywords:** next-generation sequencing, HLA, novel alleles, standardization, proficiency testing

## Abstract

Next-Generation Sequencing (NGS) has transformed clinical histocompatibility laboratories through its capacity to provide accurate, high-throughput, high-resolution typing of Human Leukocyte Antigen (HLA) genes, which is critical for transplant safety and success. As this technology becomes widely used for clinical genotyping, histocompatibility laboratories now have an increased capability to identify novel HLA alleles that previously would not be detected using traditional genotyping methods. Standard guidelines for the clinical verification and reporting of novelties in the era of NGS are greatly needed. Here, we describe the experience of a clinical histocompatibility laboratory’s use of NGS for HLA genotyping and its management of novel alleles detected in an ethnically-diverse population of British Columbia, Canada. Over a period of 18 months, 3,450 clinical samples collected for the purpose of solid organ or hematopoietic stem cell transplantation were sequenced using NGS. Overall, 29 unique novel alleles were identified at a rate of ∼1.6 per month. The majority of novelties (52%) were detected in the alpha chains of class II (HLA-DQA1 and -DPA1). Novelties were found in all 11 HLA classical genes except for HLA-DRB3, -DRB4, and -DQB1. All novelties were single nucleotide polymorphisms, where more than half led to an amino acid change, and one resulted in a premature stop codon. Missense mutations were evaluated for changes in their amino acid properties to assess the potential effect on the novel HLA protein. All novelties identified were confirmed independently at another accredited HLA laboratory using a different NGS assay and platform to ensure validity in the reporting of novelties. The novel alleles were submitted to the Immuno Polymorphism Database-Immunogenetics/HLA (IPD-IMGT/HLA) for official allele name designation and inclusion in future database releases. A nationwide survey involving all Canadian HLA laboratories confirmed the common occurrence of novel allele detection but identified a wide variability in the assessment and reporting of novelties. In summary, a considerable proportion of novel alleles were identified in routine clinical testing. We propose a framework for the standardization of policies on the clinical management of novel alleles and inclusion in proficiency testing programs in the era of NGS-based HLA genotyping.

## 1 Introduction

The history of HLA typing in transplantation began with serology-based techniques such as complement-dependent cytotoxicity assays (Middleton, 2005). This was followed by molecular platforms such as the Reverse Sequence-Specific Oligonucleotides typing (RSSO) and Sanger sequencing which continue to be the main typing methods in many HLA laboratories. In recent years, Next-Generation Sequencing (NGS) has emerged as an effective and powerful HLA typing platform. One of the major advantages of NGS is its ability to generate high-resolution genotypes across the 11 classical HLA genes at high throughput and low cost (Hosomichi et al., 2015), which enables many HLA genes to be sequenced at full length. Depending on the NGS assay, many discriminating single nucleotide polymorphisms (SNPs) between allele combinations can now be phased, thereby reducing the occurrence of cis/trans ambiguities. These collective improvements have enabled NGS to identify novel sequences at a greater capacity compared to traditional molecular methods. New alleles are discovered at a record pace in recent years, with approximately 6,000 novel alleles on average being added each year to the Immuno Polymorphism Database-Immunogenetics/HLA (IPD-IMGT/HLA Database), the official database for HLA alleles (Barker et al., 2023; IPD-IMGT/HLA Database). Much of this phenomenon can be accredited to the widespread use of NGS for HLA genotyping (Barker et al., 2023).

Currently, there is limited guidance on the best practice for the evaluation and verification of novel alleles. In addition, how to report novel alleles clinically and the level of information that should be conveyed to the end-user is unclear. The American Society for Histocompatibility and Immunogenetics (ASHI), the governing body for accredited clinical transplant laboratories, also does not currently have a Proficiency Testing (PT) Program to evaluate the ability of accredited laboratories to identify novel alleles accurately (Proficiency Testing Program).

In addition to clinical reporting, histocompatibility laboratories also play an important role in contributing to the IPD-IMGT/HLA database. While this official catalogue for documented HLA alleles is essential for the function of clinical histocompatibility by ensuring accurate and comprehensive interpretation of patient data, it is unknown the proportion of clinical HLA laboratories that routinely submit novel alleles to IPD-IMGT/HLA and potential barriers that might impede this process.

In this study, we describe the experience of an ASHI-accredited laboratory’s use of NGS and its management of novel alleles in an ethnically diverse population. In addition, we summarize the results of a nationwide survey that describes the local practice of all Canadian histocompatibility laboratories that utilize NGS for clinical HLA genotyping. Based on these results, we advocate for the standardization of practice and propose a practical framework to facilitate the management and reporting of novel alleles and a streamlined mechanism to incorporate these alleles into established international HLA databases.

## 2 Materials and methods

### 2.1 Study cohort

The study cohort included all solid organ (kidney, heart, lung, liver, and pancreas) and hematopoietic stem cell transplant candidates and donors who underwent HLA genotyping at the Vancouver General Hospital Immunology Laboratory, the provincial reference laboratory for transplantation in British Columbia, Canada. All patients and donors were sequenced by Next-Generation Sequencing (NGS) as part of the routine workflow for transplant assessment. This study was approved by the UBC Research Ethics Board (#H22-03627).

### 2.2 DNA extraction

Whole blood was extracted using the EZ1 DNA Blood 350 µL Kit (Catalog 951054) or QIAsymphony DSP DNA Mini Kit (192) (Catalog 937255) (Qiagen, Germany). Both methods use magnetic beads to isolate DNA from leukocytes where the resulting DNA is eluted in water or a buffer. DNA was quantified using the Qubit Fluorometer (ThermoFisher, United States) and diluted to 10–35 ng/μL for sequencing.

### 2.3 NGS

Samples were sequenced with the Holotype HLA Kit version 2 (Omixon, Budapest). DNA underwent PCR amplification of the 11 classical HLA genes (HLA-A, -B, and -C; DRB1, -DRB3/4/5, -DQA1, -DQB1, -DPA1, and -DPB1). HLA-A, -B, -C, -DPA1, -DQA1, and–DQB1 were sequenced in their entirety (i.e. 5′UTR–3′UTR), HLA-DRB1, -DRB3, and -DRB4 were sequenced from partial intron 1 to partial intron 4, and HLA-DRB5 and -DPB1 were sequenced from partial intron 1 to partial 3′-UTR. Amplicons were prepared for sequencing by enzymatic fragmentation, end-repair, and ligation of adaptors provided by the kit. Prepared libraries were sequenced using MiSeq Sequencer with the 300-cycle MiSeq Reagent Cartridge (Illumina, California, United States).

Sequence data were first routinely analyzed using HLA Twin (Omixon) (Omixon, Budapest, Hungary) versions (v) 4.3.0 and 4.4.1 with IPD-IMGT/HLA data versions 3.39, 3.43, and 3.45. All samples with novel mutations were re-analyzed on HLA Twin v4.8.1 using IPD-IMGT/HLA v3.51 prior to submission into the IPD-IMGT/HLA database.

### 2.4 Identification of novel alleles

Under our current clinical practice, novel alleles are identified by the NGS analysis software HLA Twin (Omixon) (Omixon, Budapest, Hungary). The software aligns the sequenced data (i.e., consensus sequence) against the reference sequences of HLA alleles in the IPD-IMGT/HLA database to identify the allele with the most similarity to the consensus sequence. When there are exon mismatch(es) between the reference sequence and the consensus sequence, this is highlighted as a novelty by the software. A novel allele is indicated by the most related reference sequence followed by a “#1” at the end of the allele name. For example, the A*03:452 novelty was identified as A*03:05:01#1 at the point of routine analysis.

This study focused on the identification of alleles with novelties in exons. In alleles where intronic novelties co-occurred with exonic novelties, the intronic SNPs were also flagged and reported as part of the submission to the IPD-IMGT/HLA database. All samples with novel alleles passed the quality control (QC) metrics on the HLA Twin Software. As per our laboratory’s standard operating procedure, all novel alleles were further verified by another ASHI-accredited HLA laboratory (HLA Typing Laboratory, Halifax, Nova Scotia, Canada) using an alternate NGS HLA kit, AllType FASTplex NGS (OneLambda, United States) and NGS platform, Ion Torrent (Thermofisher, United States).

### 2.5 RSSO and real-time PCR (RT-PCR)

In addition to confirmatory testing using an alternative NGS-based assay, RSSO, and RT-PCR were also performed for a proportion of samples during routine testing. RSSO was performed with the LABType SSO and XR kits (OneLambda, United States), where DNA was amplified at key regions of the HLA genes. Amplicons were then denatured and hybridized to sequence-specific oligonucleotides on beads. The bound amplicon was detected using PE-conjugated streptavidin and beads were ran through the LABScan3D (OneLambda, United States) flow analyzer. Reaction patterns to determine HLA typing were performed in HLA Fusion (Onelambda, United States). RT-PCR was performed using the LinkSeq PCR Typing Kits (OneLambda, United States) where sequence-specific primers amplified regions of DNA, and fluorescent reaction patterns were detected using the LightCycler 480 System (Roche, Canada) to determine HLA typing at low to intermediate resolution. Reaction patterns were analyzed on SureTyper for HLA (OneLambda, United States).

### 2.6 Submission to GenBank and IPD-IMGT/HLA

As per IPD-IMGT/HLA submission guidelines, novel sequences were first submitted to GenBank using BankIt (BankIt). Once an accession number was assigned, novel alleles were then submitted to IPD-IMGT/HLA for confirmation and documentation. The novel alleles were officially assigned by the WHO Nomenclature Committee for Factors of the HLA System in September–October 2022. This follows the agreed policy that, subject to the conditions stated in the most recent Nomenclature Report (Marsh et al., 2010), names will be assigned to new sequences as they are identified. Lists of such new names will be published in the following WHO Nomenclature Report.

### 2.7 Ethnicity determination

The majority of ethnicities (76%) of the study cohort were self-reported. When self-reported ethnicity was not available (24%), it was inferred based on the most probable predicted ethnicity using Haplostats, based on HLA-A, -C, -B, -DRB3/4/5, -DRB1, and -DQB1 associations (National Marrow Donor Program).

### 2.8 Analysis of non-synonymous/missense mutations

To assess the potential impact of non-synonymous mutations on the HLA protein phenotype, changes in the type and location of the modified amino acid were analyzed and assigned a score. First, variables that were used for the analysis included whether the mutation resulted in an amino acid change. As this was true for all non-synonymous mutations, all novelties with a missense mutation were assigned a “+”. Next, the basic characteristics of the original and novel amino acids were compared based on the properties listed in Supplementary Table S1 (Sanvictores and Farci, 2022). If there was a change in the basic properties (non-polar (aromatic/aliphatic) versus polar (basic, acidic, uncharged) of the original residue, an additional “+” was assigned. If the novel mutation led to an altered amino acid in an antigen-binding site (i.e., exons 2 and 3 for class I and exon 2 for class II), another “+” was assigned. A “+” was further assigned if the mutation affected an HLA eplet as defined by the HLA Eplet Registry (HLA Eplet Registry). An eplet was defined as the critical amino acids residing within 3.0–3.5 Angstrom radius that constitute an HLA epitope considered essential for antibody-binding and specificity (Duquesnoy, 2006). To assess for potential changes to eplets, we evaluated whether the novel mutation occurred at a position of a known eplet that is expressed by the original allele or if the mutation introduced a new eplet. Combining all of the scores assigned above, each missense novelty can have a minimum of one “+” designation to four (i.e., “++++”).

## 3 Results

### 3.1 Description of novel sequences

A total of n = 3,450 clinical samples were sequenced using NGS to derive high-resolution HLA genotypes from 1 January 2021–1 July 2022 (18 months), averaging approximately 48 samples per week. During this timeframe, 29 unique novel alleles (Table 1) were identified in 41 samples (Supplementary Table S2). Six of the novel alleles were detected in multiple patient samples due to the sequencing of related donors and patients, which resulted in the same novel mutation being identified in related samples. For example, the novelty DQA1*05:05:16 was identified in two samples, one from a kidney patient and one from their related offspring donor. In another case, the novel allele DQA1*01:01:09:02 was identified in seven samples, where one was from a patient candidate for a bone marrow transplant, and three were from related siblings. Interestingly, the remainder three samples were not related to the patient where two were derived from matched unrelated donors and another was from an unrelated kidney patient. On average, two samples with a novel mutation were identified per month. When analyzed by unique novel mutations, 1.6 novelties were identified per month.

**TABLE 1:** The unique novel HLA alleles identified in the study. Details of the novel mutations identified in exons are described here, including the type of mutation, exon location, the corresponding location on the protein, the nucleotide change, and the IPD-IMGT/HLA genomic position of the mutation.

Novel allele	Type of mutation	Exon	Location on protein	Nucleotide change	IMGT genomic position
A*03:452	Missense	Exon 3	Antigen-binding site, α2	A > C	873
A*26:203*	Missense	Exon 1	Leader peptide	G > C	28
B*15:675	Missense	Exon 1	Leader peptide	C > T	47
B*48:01:12	Silent	Exon 3	Antigen-binding site, α2	C > T	736
B*48:55	Missense	Exon 4	α3 extracellular arm	A > G	1701
B*56:88*	Missense	Exon 3	Antigen-binding site, α2	A > C	793
C*05:277	Missense	Exon 7	Cytoplasmic tail	G > C	2721
C*07:1041	Missense	Exon 2	Antigen-binding site, α1	C > A	256
C*07:1043	Missense	Exon 2	Antigen-binding site, α1	T > G	385
DPA1*01:03:38:02*	Silent	Exon 1	Leader peptide	C > T	51
DPA1*01:03:45	Silent	Exon 3	α2 extracellular arm	C > T	4506
DPA1*01:106*	Missense	Exon 1	Leader peptide	G > A	5
DPA1*01:136	Missense	Exon 3	α2 extracellular arm	A > G	4463
DPA1*01:137N	Nonsense	Exon 1	Leader peptide	C > T	79
DPA1*01:60*	Missense	Exon 4	Transmembrane/cytoplasmic tail	C > T	4901
DPA1*02:02:13	Silent	Exon 4	Transmembrane/cytoplasmic tail	T > C	4876
DPA1*02:96	Missense	Exon 4	Transmembrane/cytoplasmic tail	A > T	4850
DPB1*1088:01*	Missense	Exon 4	Transmembrane	A > G	9701
DQA1*01:01:09:02*	Silent	Exon 1	Leader peptide	C > G	36
DQA1*01:02:15	Silent	Exon 1	Leader peptide	C > T	48
DQA1*01:02:16	Silent	Exon 2	Antigen-binding site, α1	C > A	3976
DQA1*01:04:08	Silent	Exon 3	α2 extracellular arm	C > T	4548
DQA1*02:01:15Q	Silent	Exon 2	Antigen-binding site, α1	C > T	3809
DQA1*04:01:07	Silent	Exon 1	Leader peptide	C > T	48
DQA1*05:05:16	Silent	Exon 1	Leader peptide	T > A	69
DRB1*14:249	Missense	Exon 2	Antigen-binding site, β1	C > T	8091
DRB1*14:54:12*	Silent	Exon 3	β2 extracellular arm	A > G	10752
DRB5*01:130	Missense	Exon 3	β2 extracellular arm	G > T	10808
DRB5*02:37	Missense	Exon 3	β2 extracellular arm	A > G	10732

An asterisk (*) indicates an allele that was detected as novel at the time of analysis but upon submission into IPD-IMGT/HLA, had already received an official name by an independent laboratory.

Among the 41 samples identified with a novel mutation in this study, almost half of the samples (n = 20, 49%) were detected in patients (n = 11, 27%) or donors (n = 9, 22%) considered for solid organ transplantation. Another 20 samples were derived from patients (n = 3, 7%) considered for a bone marrow transplant and their potential donors (n = 17, 41%). One additional sample with a novelty was identified in a patient tested for genetic disease association of ankylosing spondylitis.

**FIGURE 1:**
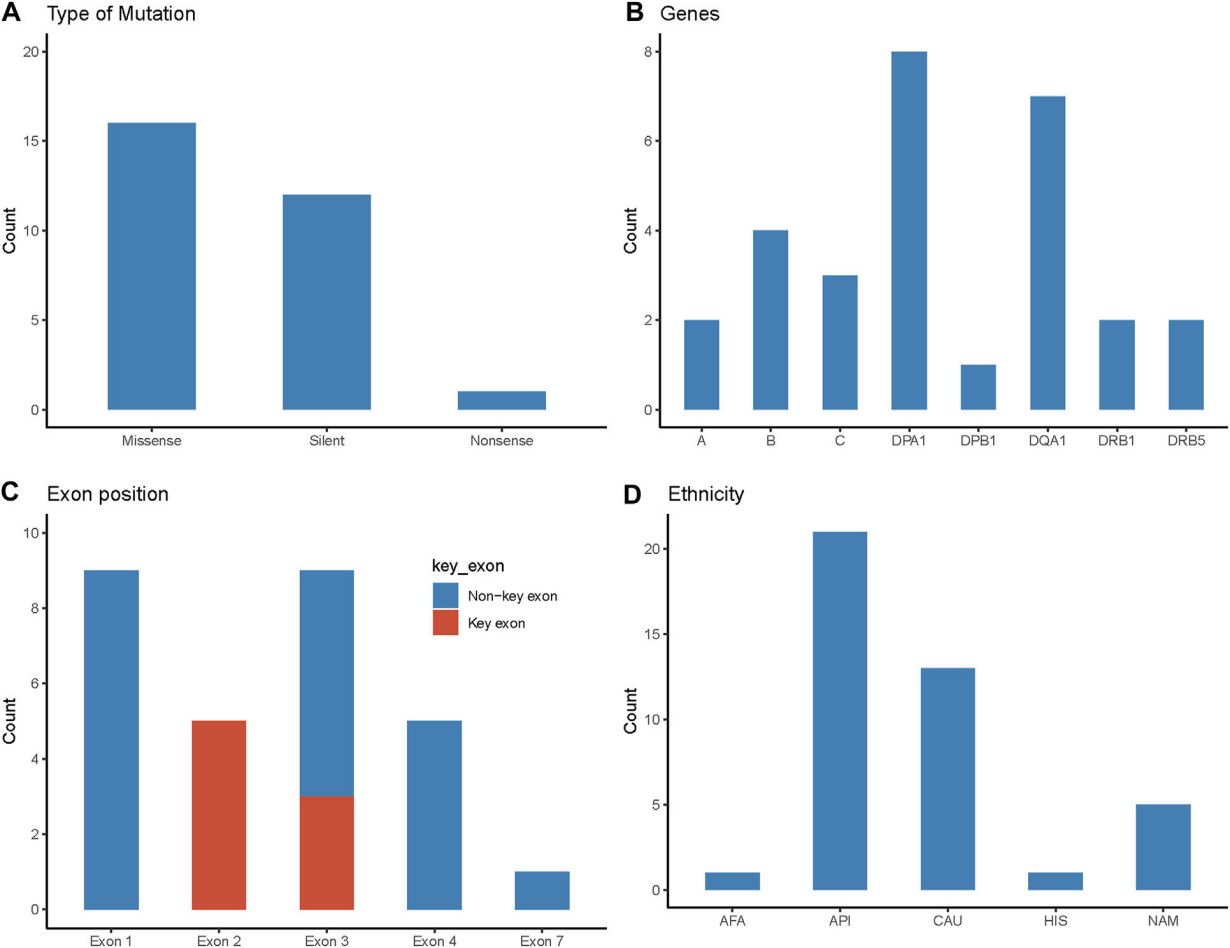
Characteristics of novel mutations identified in the study cohort. **(A)** Type of the novel mutations identified in the study and their frequency. **(B)** The HLA gene locus where the novel alleles were identified. **(C)** The exon position of novel mutations. Key exon represents the region that encodes the antigen-binding site of the HLA protein. Exons 2 and 3 are key exons for class I HLA proteins and exon 2 is the key exon for class II proteins. **(D)** Ethnicity of patients with novel mutations (n = 41). AFA, African American; API, Asian Pacific Islander; CAU, Caucasian; HIS, Hispanic; NAM, Native American.

All novelties reported in this study were single nucleotide polymorphisms (SNPs): 16 (55%) were missense mutations resulting in a non-synonymous amino acid change; 12 (41%) were silent mutations with no amino acid change; and one novelty (3%) resulted in a stop codon (i.e., nonsense) (Figure 1A).

In this study cohort, a novel allele was identified in all 11 HLA classical genes, except for HLA-DRB3, -DRB4, and -DQB1 (Figure 1B). Of the 29 novel alleles, HLA-DPA1 had the most novelties with eight sequences, followed by HLA-DQA1 with seven. HLA-B had four novel sequences, followed by HLA-C with three novelties. HLA-A, -DRB1, and -DRB5 had two novel sequences each. Lastly, HLA-DPB1 had one novel allele.

Eight novelties (28%) identified in this study occurred in the key exons that encode the antigen-binding site (i.e., exons 2 and 3 for class I and exon 2 for class II) (Figure 1C). Nine (31%) of the mutations occurred in exon 1 which encodes the leader peptide. Another nine novelties (31%) occurred in exon 3, where three were considered key exon changes (as stated above for class I) and six were non-key exons encoding the alpha-2 or beta-2 region in HLA class II. Five (17%) novel alleles were due to exon 4 mutations, where one was found in HLA class I (HLA-B*48:55) which encodes alpha-3, and the other four belonged to HLA class II, encoding the transmembrane/cytoplasmic regions. Lastly, there was one novelty (3%) in exon 7 (HLA-C*05:277), encoding for the cytoplasmic region of the protein.

The ethnic makeup of patients/donors with novelties was predominantly Asian or Pacific Islander (51%), followed by Caucasian (32%), Native American (12%), Hispanic (2%), and African American (2%). These proportions were similar to patients/donors of only self-reported ethnicities as well, where the proportions were Asian or Pacific Islander (52%), Caucasian (32%), Native American (13%), and African American (3%). These compositions were noticeably different from the general Canadian and British Columbian population, in which European origins comprise 53% and 60%, respectively, of self-reported ethnicity (compared to 32% in this cohort) with smaller proportions of minorities (Statistics Canada).

Non-synonymous/missense mutations where a single base pair alteration resulted in an amino acid change were present in 16 novel alleles. We studied the potential effect of the missense mutation on the protein’s phenotype by the location and change in the basic properties of the original and new amino acid (Table 2). In addition, we assessed if the mutation had a potential effect on antibody-binding by evaluating if it occurred in the same position as a known eplet. For example, the B*56:88 novelty was designated “++++” due to the mutation resulting in a change in amino acid (“+”) between residues of different basic chemical properties (original leucine was nonpolar aliphatic and the novel phenylalanine was nonpolar aromatic) (“+”), and that this mutation occurred in the antigen-binding site (“+”) and also affected a known eplet (“+”). This is in contrast to the C*05:277 novelty where a polar uncharged cysteine was changed to a serine which is also polar uncharged. This mutation occurred in the cytoplasmic tail and did not affect antibody binding (i.e., no eplet). Based on this analysis, the C*05:277 was marked with just one “+” as a potential effect on the protein’s phenotype. The amino acid alteration score of other non-synonymous missense mutations identified in this study are shown in Table 2.

**TABLE 2:** Novel alleles resulting in non-synonymous/missense mutations. The potential effect of missense mutations was assessed by comparing the novel amino acid to the original amino acid through differences in location, amino acid characteristics (Supplementary Table S1), and impact on the associated eplet (HLA Eplet Registry).

Novel allele	Location on protein	IMGT-IPD/HLA codon position	Original a.a.	Basic properties of original a.a.	Novel a.a.	Basic properties of novel a.a.	Potential effect on protein	Variables that constitute “Potential effect on HLA protein”			
								A.a. change	Change in a.a. basic properties	Occurs in antigen-binding site	Affects an eplet
A*03:452	Antigen-binding site, α2	144	Lys	Basic	Gln	Polar uncharged	++++	*+*	*+*	*+*	*+*
A*26:203	Leader peptide	−15	Val	Nonpolar aliphatic	Leu	Nonpolar aliphatic	+	*+*			
B*15:675	Leader peptide	−9	Ala	Nonpolar aliphatic	Val	Nonpolar aliphatic	+	*+*			
B*48:55	Extracellular arm, α3	228	Thr	Polar uncharged	Ala	Nonpolar aliphatic	++	*+*	*+*		
B*56:88	Antigen-binding site, α2	116	Leu	Nonpolar aliphatic	Phe	Nonpolar aromatic	++++	*+*	*+*	*+*	*+*
C*05:277	Cytoplasmic tail	340	Cys	Polar uncharged	Ser	Polar uncharged	+	*+*			
C*07:1041	Antigen binding site, α1	17	Arg	Basic	Ser	Polar uncharged	++++	*+*	*+*	*+*	*+*
C*07:1043	Antigen binding site, α1	60	Trp	Nonpolar aromatic	Gly	Nonpolar aliphatic	+++	*+*	*+*	*+*	
DPA1*01:106	Leader peptide	−30	Arg	Basic	His	Basic	+	*+*			
DPA1*01:136	Extracellular arm, α2	149	His	Basic	Arg	Basic	+	*+*			
DPA1*01:60	Transmembrane/cytoplasmic tail	224	Arg	Basic	Trp	Nonpolar aromatic	++	*+*	*+*		
DPA1*02:96	Transmembrane/cytoplasmic tail	213	Ile	Nonpolar aliphatic	Phe	Nonpolar aromatic	++	*+*	*+*		
DPB1*1088:01	Transmembrane	194	Gln	Polar uncharged	Arg	Basic	++	*+*	*+*		
DRB1*14:249	Antigen-binding site, β1	6	Arg	Basic	Cys	Polar uncharged	++++	*+*	*+*	*+*	*+*
DRB5*01:130	Extracellular arm, β2	160	Met	Nonpolar aliphatic	Ile	Nonpolar aliphatic	+	*+*			
DRB5*02:37	Extracellular arm, β2	150	Asn	Polar uncharged	Ser	Polar uncharged	+	*+*			

**FIGURE 2:**
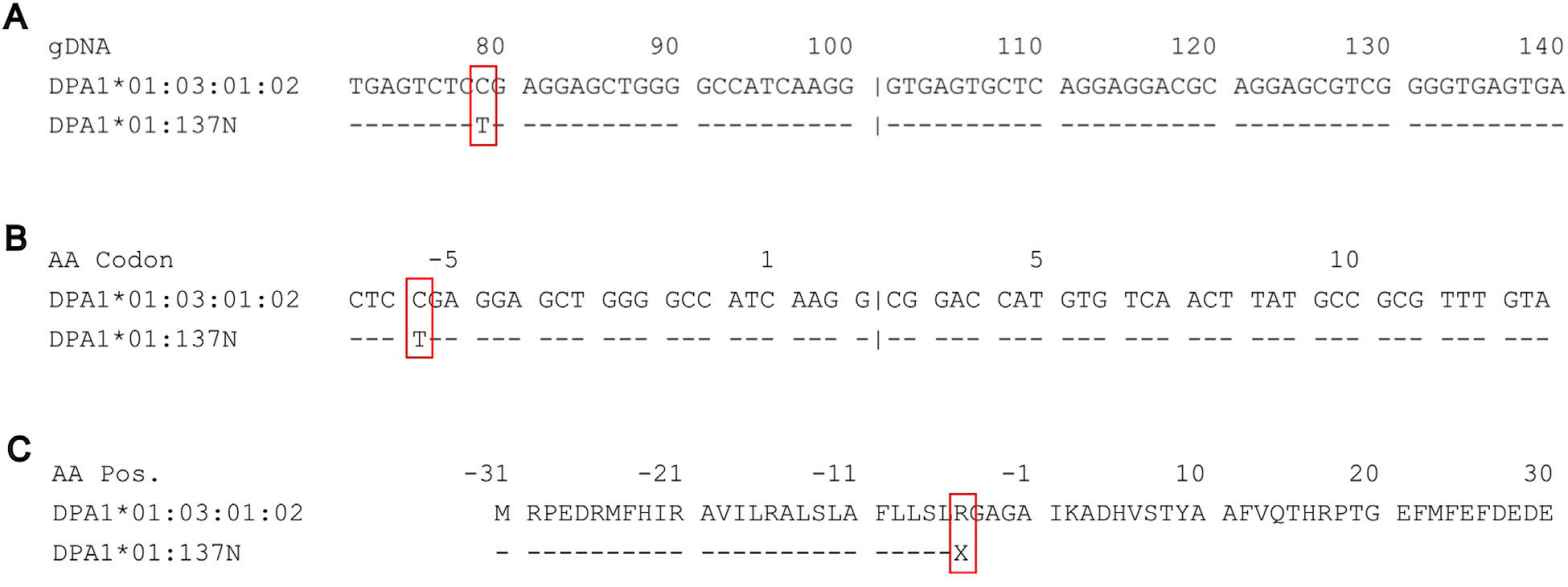
Sequence alignments on IPD-IMGT/HLA of DPA1*01:03:01:02 against the novel nonsense mutation DPA1*01:137N. **(A)** Genomic sequence alignment of partial exon 1 and intron 1 of DPA1*01:03:01:02 (top) against the novel allele DPA1*01:137N (bottom). A novel mutation at IMGT genomic position 79 from C (cytosine) to T (thymine). **(B)** Coding sequence alignment of partial exon 1 and exon 2, demonstrating a change in the −5 codon CGA to TGA. **(C)** Amino acid sequence alignment of the leader peptide and partial antigen-binding site, demonstrating the nonsense mutation from R (arginine) to X (stop codon) occurring in the leader peptide.

In one sample, we identified a nonsense mutation in exon 1 of DPA1 (HLA-DPA1*01:137N) (Figure 2). The mutation led to a change from cytosine (IMGT genomic position 79) to thymine (Figure 2A), resulting in the codon change of CGA to TGA (IMGT codon position −5) (Figure 2B), which encodes a stop codon at the point of the 5′ leader peptide (Figure 2C).

Many novelties included nucleotide changes that did not result in an amino acid change (12 unique silent novel sequences). However, for DQA1*02:01:15Q, although the exon novelty was a silent mutation, there was also an additional SNP in intron 2 (IMGT genomic position 4027) that may affect splicing, resulting in questionable (Q) expression.

### 3.2 Discrepant results for patients with hematological malignancies

We identified two patients diagnosed with acute myeloid leukemia that had discrepant HLA typing between DNA collected from different specimens (Supplementary Table S2). The first patient (VGH050), based on DNA extracted from peripheral blood, was found to have a guanine insertion in exon 2 of B*15:01:01:01, resulting in a novel frameshift mutation when sequenced using NGS. When re-testing on RSSO and RT-PCR, both assays yielded a “normal” B*15:01:01:01 allele. A buccal swab was then collected to investigate the possibility that the novelty was the result of cancerous mutation in the malignant cell line. Indeed, the buccal-derived DNA when sequenced using NGS yielded a “normal” B*15:01:01:01 allele without reads to support the previously observed guanine insertion in exon 2.

In a similar pattern, a second patient’s (VGH049) initial DNA sample extracted from peripheral blood resulted in a novel SNP mutation (G > T) in exon 3 of A*02:06:01:01. However, a buccal swab was then collected which resulted in a “normal” A*02:06:01:01 without the novel mutation. In both cases, novel alleles were only observed in DNA extracted from patients’ peripheral blood and not the buccal cells, indicating the typing discrepancy was most likely attributed to abnormal mutations derived from circulating malignant cells. Thus, a typing based on germline cells is always required for patients with hematological malignancies to account for cancer cells yielding a different HLA genotype from the patient’s actual typing, especially if a novel mutation was detected. This is also a requirement for submission into the IPD-IMGT/HLA database.

### 3.3 Confirmation of novel alleles by different molecular assays

As described in the examples above, RSSO and RT-PCR did not confirm any of the novelties identified by NGS in our cohort. In most cases, these molecular methods yielded the “normal” allele type most related to the novel sequence but were not able to identify the mutation. However, in one sample (VGH029), the confirmatory RSSO resulted in a completely inaccurate result (Supplementary Figure S1). NGS identified a novel missense mutation for HLA-B*56:02:01:01 due to a nucleotide change of adenine to cytosine (IMGT genomic position 793). This resulted in the amino acid change of leucine to phenylalanine (IMGT codon position 116). At the time, the NGS analysis software assigned a typing of B*40:01 + B*56:02:01:01#1. Re-testing the sample on RSSO yielded a discrepant result for both normal and novel alleles: B*40:36 + B*55:08. Comparing the sample reaction pattern to what was expected of B*40:01 + B*56:02, two beads were unexpectedly positive and one was negative. Further investigation showed that all three probes bound to the region in which the novel mutation occurred, and the presence of the mutation resulted in unexpected reaction patterns to yield inaccurate typing. The new allele name for this novel sequence was B*56:88.

To ensure the accuracy of the novel sequences, all samples were re-tested using an alternate NGS sequencing platform in an external ASHI-accredited sister laboratory. All samples were concordant in identifying the same novel sequence, in addition to having complete concordance at the remaining HLA loci.

### 3.4 Investigation of novel alleles and reporting to the clinical team

During routine testing, all alleles with novel exon mutations underwent further investigation prior to clinical sign out. The mutations were evaluated for their effect(s) on the coding amino acids (e.g., missense/non-synonymous, silent/synonymous, nonsense/truncated protein, insertion/frameshift) as well as the exon location and if the mutation occurred in the antigen-binding site.

Novel alleles were communicated to the clinical team through a custom comment in the HLA typing case reports. The comments were written by the laboratory director and conveyed the presence of a novel mutation and relevant details of the mutation, including the nucleotide and amino acid changes, exon location, and if it occurred in the antigen-binding site.

### 3.5 Submission to IPD-IMGT/HLA

At the point of submitting the novel sequences to the IPD-IMGT/HLA database (i.e., September 2022), eight of the novelties were already registered and given an official name (alleles ending with an asterisk in Table 1). The remaining 21 novel sequences were submitted together. Many of the key requirements for novel allele submission outlined by IPD-IMGT/HLA, such as bi-directional sequencing; typing at HLA-A, B, and DR loci; and a minimum of key exon sequencing (IPD-IMGT/HLA Database), were fulfilled by the commercial NGS kit and software used in our laboratory. However, there were additional requirements beyond the typical scope of HLA genotyping in the clinical laboratory. One main criterion included obtaining an accession number for every novel allele identified. To do this, the novel sequences require submission first to any of the following three public sequence repositories: DDBJ (DNA Data Bank of Japan) (DDBJ), ENA (European Nucleotide Archive) (ENA Browser), or GenBank (BankIt).

**FIGURE 3:**
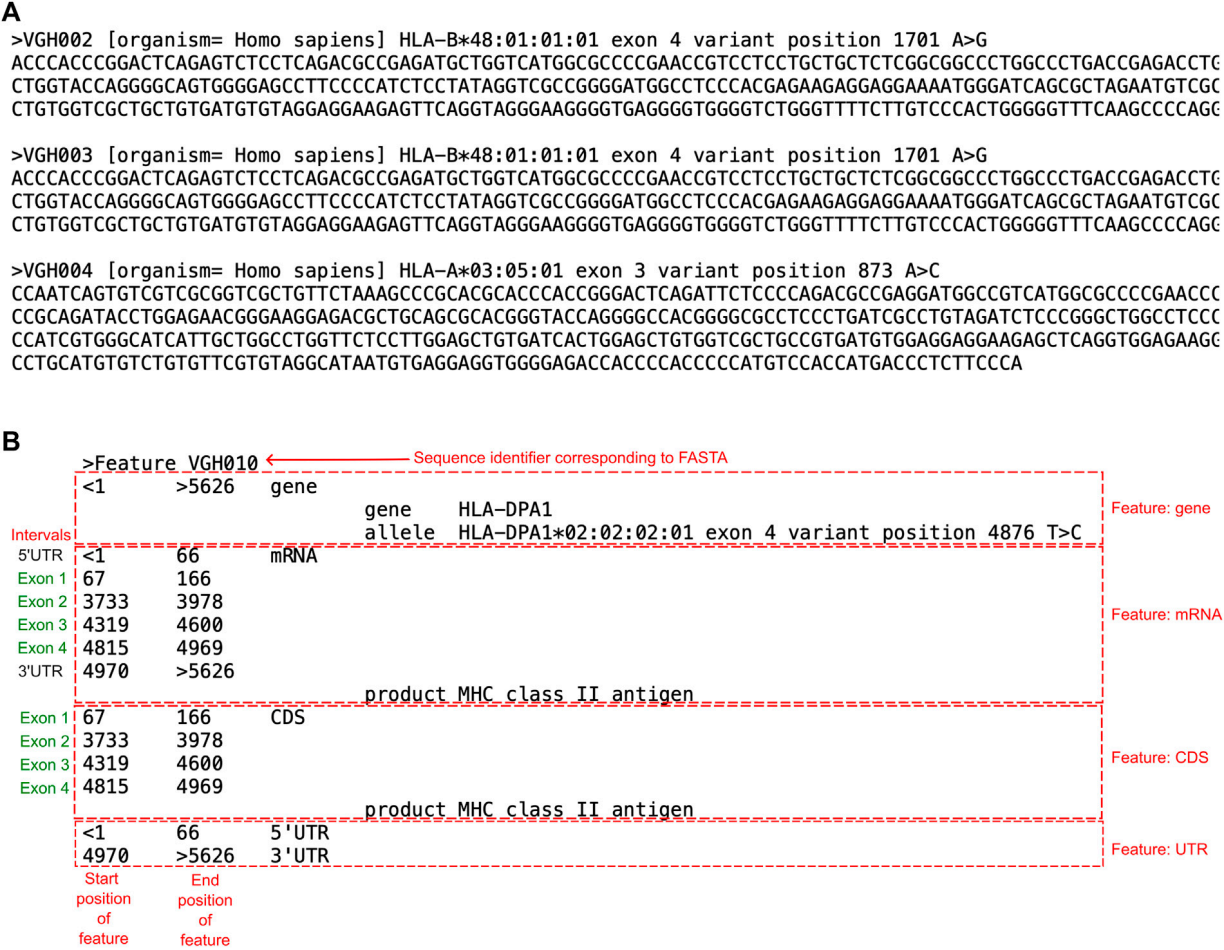
Examples of the FASTA and Feature Table File required for IPD-IMGT/HLA submission. **(A)** A screenshot of three novel allele entries submitted to GenBank as a FASTA file. Each entry begins with a “>”, followed by the “SeqID” which acts as the identifier (ex. VGH002). This is followed by the organism the DNA was extracted from in brackets, and then a descriptor of the entry. In the next line is the sequence of the entry, which includes the novel mutation. **(B)** Example of a Feature Table File used to submit into GenBank. Each entry in a Feature Table File began with a “>”, followed by “Feature” and the sequence identifier that corresponded to the FASTA file. The corresponding text described details of features regarding the submitted sequence, such as the gene, mRNA, or coding sequence (CDS). For every feature, the position of when the feature begins and ends within the corresponding FASTA file is indicated. For the “gene” feature, the gene begins at nucleotide 1 of the FASTA file and ends at nucleotide 5626. The “mRNA” feature, the UTR and exon regions are indicated. The “CDS” feature indicates only exon positions. The 5′UTR and 3′UTR represent the untranslated regions.

GenBank’s submission tool BankIt was used to obtain accession numbers for the submitted samples with novelties. A wizard guided the submission process where multiple sequences were included in one submission. Information required for this process included the sequences of the novel alleles. In this study, we uploaded a single master FASTA file that combined all novel allele FASTA files exported from the analysis software. One entry consisted of a “SeqID” acting as a sample identifier, where the organism was included with a descriptor “*Homo sapiens*”, followed by a brief description of the sequence (Figure 3A). This was immediately followed by the actual sequence containing the novel mutation in the next line.

The sequences were annotated using a “feature table file” (Figure 3B). The feature table was a plain text file that corresponded to each sequence in the FASTA file using the “Sequence identifier”. The table annotates “features” of the FASTA, including the sequenced gene, mRNA, and coding sequence (CDS) relevant to the submitted sequence. Within each feature are annotations of nucleotide positions of intervals relevant to the feature. For example, the mRNA feature was divided into the UTRs and exons. Each FASTA corresponding to a novel sequence had its own feature table. Upon submission of the FASTA and feature table files, GenBank provided accession numbers for the sequences.

The novel sequences were then submitted to IPD-IMGT/HLA to request an official naming into the database. This process required a written description of the mutation observed, including its position, type of mutation, and any phenotypic changes. An example was “VGH002 has 1 nt change from B*48:01:01:01 at nt 1,701 where A > G (codon 228 ACT > GCT), resulting in a coding change 228 Thr is changed to Ala.” The IPD-IMGT/HLA website provided a tool that can help annotate sequence features for class I sequences (but not for class II). However, as a feature table of annotations was required to obtain an accession number from GenBank, this information was already determined at the point of IPD-IMGT/HLA submission. The submission form also included sample demographics if available, such as ethnicity and sex. Successful submissions resulted in an official report provided by IPD-IMGT/HLA and a new allele name for each reported novelty.

There was one submission for a DRB3 allele that could not be officially named because the novel sequence contained a silent variant that required complete coding sequence data to differentiate it from other possible proteins. The sequencing kit used in our laboratory covered only exon 2 to exon 4 and thus could not rule out potential variants in exons 1, 5, 6 for DRB3 variants. This sample (VGH025) was excluded from the final study cohort (Supplementary Table S2).

### 3.6 Canadian survey

We conducted a nationwide survey including all 16 Canadian HLA Laboratories serving hematopoietic stem cell transplant and/or solid organ transplant programs to evaluate their experience with novel allele detection and reporting using NGS (Table 3). Overall, 10/16 (63%) laboratories employed NGS technology as their primary genotyping tool. A variety of kits were used to prepare sequencing libraries: the top two were AllType FastPlex (OneLambda) (n = 3, 30%) and NGSgo 11 (GenDx) (n = 3, 30%), followed by Holotype (Omixon) (n = 1, 10%), Nanotype (Omixon) (n = 1, 10%), Mflex 11 Typing Kit (Mia Fora) (n = 1, 10%), and in-house reagents (n = 1, 10%). The leading sequencing platform was the MiSeq (n = 6, 60%), followed by the MiniSeq (n = 2, 20%). One laboratory used the Ion Torrent system while another adopted the emerging Nanopore Minion which delivered real-time rapid sequencing with long reads.

**TABLE 3:** Canadian survey on novel allele detection. The results of the ten Canadian HLA laboratories that use NGS for as the main sequencing platform.

	Count	Proportion (%)
Commercial Kit		
AllType FastPlex	3	30
Omixon Holotype	1	10
Omixon Nanotype	1	10
NGSgo 11 loci from GenDx	3	30
In-house reagents with GenDx reflex kit	1	10
Mia Fora Mflex 11 Typing Kit	1	10
Sequencing Platform		
MiniSeq	2	20
Nanopore Minion	1	10
MiSeq	6	60
Ion Torrent	1	10
Approximate number of novelties detected		
None	1	10
Less than two a month	4	40
More than two a month	4	40
Other	1	10
Other reason	• Unknown as there are regions not sequenced due to a lack of primer coverage	
*The following applies to only laboratories that have detected novel alleles*		
Confirm novel alleles		
Yes	3	33
No	1	11
Selective confirmation	5	56
	• Depends on mutation/recombination	
	• Depends on patient category, such as for patients with leukemia or when submitting to IPD-IMGT/HLA	
	• Depends on the position of the mutation	
	• Occasionally (no criteria listed by respondent)	
Novel allele investigation		
No additional investigations	0	0
Location of novelty (ex. exon, intron, antigen binding site)	9	100
Type of mutation (ex. missense, silent, insertion, deletion)	8	89
Splicing	6	67
Other	1	11
Other reasons	• Identify parent alleles for recombination	
Reporting of novel alleles		
The most similar allele or G/P group without additional comments	0	0
The most similar allele or its G/P Group with comment	3	33
The most similar allele or its G/P group with comment only if it has a reasonable effect on the protein	4	44
The custom allele with novelty noted	1	11
Antigen equivalent if allele cannot be reported as a G/P group	1	11
Does your laboratory submit novel alleles to the IPD-IMGT/HLA database?		
Yes	7	78
No	2	22

All but one laboratory routinely detected novel HLA alleles with their sequencing platform. One other laboratory also reported never detecting novelties but this was likely attributed to a lack of primer coverage resulting in the inability to rule out novelties occurring in the unsequenced regions. Of the remaining, four laboratories (40%) reported a novel allele detection rate at less than two per month, while another four (40%) report detecting two or more per month.

There was a mixed response from the laboratories in their protocol for the verification of novel alleles and the method(s) utilized. The majority of the laboratories would only selectively retest patient samples depending on the novelty or patient/donor type. For example, one surveyed laboratory reported only confirming novelties for patients requiring a bone marrow transplant or when submitting to IPD-IMGT/HLA. Others reported confirmatory testing only if the mutation arose in a clinically meaningful location, such as in coding region or key exons. Three laboratories routinely confirmed novel alleles with a sister laboratory using a different NGS kit. One repeated NGS typing in addition to SSP (Olerup SSP by CareDx) within their own laboratory. One center did not engage in confirmation of novel alleles when detected. Despite this heterogeneity in practice, all centers performed additional analysis to investigate the location of the mutation to inform the predicted impact of the mutation on the expressed protein product. In addition, 67% of the laboratories reported routine evaluation of the effect of nucleotide change on RNA splicing.

Further heterogeneity was observed in the reporting of novel alleles. Some centers reported the most similar allele (ex. the allele with the same sequence as the novelty except for the mutation) or the novel allele’s G/P group if it belonged to one. If the mutation arose in a key exon, one center reported the novel allele’s antigen equivalent. One laboratory reported a custom allele, where the term “NEW” was added to the second field (ex. DQA1*01:NEW). A proportion of laboratories (n = 3, 33%) included an additional comment on the report describing the novelty, whereas some only included a clinical comment if the novelty had a clinically meaningful effect on the protein (n = 4, 44%). Only two laboratories consistently submitted novel alleles into the IPD-IMGT/HLA database and five laboratories only submitted their alleles once or a limited number of times, or were just beginning to do so. Many survey respondents reported challenges in submitting novelties, including a lack of resources and time, the perceived requirement to re-sequence a second sample, and limitations of gene coverage resulting in complications in reporting.

## 4 Discussion

Next-Generation Sequencing has transformed HLA genotyping in the clinical histocompatibility laboratory with its capacity to provide high-throughput and high-resolution genotypes at the 11 classical HLA genes. One of the advantages of NGS is the ability to discover novel sequences through routine testing. Unfortunately, there is limited societal guidance on how to manage novel alleles in the clinical laboratory. Herein, we described the experience of an ASHI-accredited laboratory that uses NGS as a routine testing technology and performed a nationwide survey including the HLA laboratories in Canada to highlight important laboratory and clinical considerations in the detection and reporting of novel alleles. Based on our findings, we advocate for consensus-building and the development of best practices on novel allele management.

In the multi-ethnic population of 5 million in British Columbia, Canada, the overall rate of detecting novel mutations was approximately 1.6 per month. This frequency is comparable to the frequency observed in other Canadian laboratories and may even be considered “common” based on the standards of the Common-Intermediate and Well-Documented (CIWD) catalog (Hurley et al., 2020). Indeed, other groups have also reported on novel alleles after the adoption of NGS in clinical HLA laboratories (Shen et al., 2022; Dhuyser et al., 2023; Kouniaki et al., 2023; Liacini et al., 2023; Zhong et al., 2023). In one study, the DKMS Life Science Laboratory reported the detection of 1,919 unique novel allele sequences out of 1.4 million donors sequenced, but this frequency only represents the discovery rate in a genetically homogenous stem cell donor population (Schöfl et al., 2017).

While these data highlight the common occurrence of novel HLA alleles encountered in a HLA laboratory, there are limited guidelines on how to handle these novel sequences clinically. The World Marrow Donor Association recently published recommendations on novel allele reporting for hematopoietic stem cell transplants (Hofmann et al., 2023). The publication describes how to best communicate novel alleles between registries for unrelated donor searches, taking into consideration the mutation type (non-synonymous vs. synonymous) and location (antigen-binding site vs. outside this region). However, additional considerations are required when reporting novel alleles in the clinical setting, which might include the assessment of mutations outside the antigen-binding site (ex. splice site variants) and their potential impact on antibody-binding (ex. effect on eplet), as well as the physiochemical changes of non-synonymous mutations. To our knowledge, the only ASHI policy according to the ASHI Standards (2022) is D.5.2.5.8: “Laboratories must determine the sequences of both sense and anti-sense DNA strands if a sequence suggests a novel allele” (American Society for Histocompatibility and Immunogenetics, 2023). For IPD-IMGT/HLA, there are further requirements for allele submission, such as novel sequences identified by NGS must be completely phased, but the remaining standards may pertain more to molecular techniques that are not NGS-based (e.g., Sanger Sequencing) (IPD-IMGT/HLA Database). This lack of standardization has resulted in a wide variation of practice and uncertainty in the discovery and documentation of novel alleles by histocompatibility laboratories.

There is no official requirement by ASHI to verify novel sequences identified by NGS. Additionally, there is ambiguity on whether a novel sequence should be confirmed and if so, what method is most appropriate. For example, RSSO and RT-PCR were not able to detect the novel sequences accurately at our center. Instead, the results were either the most similar allele or even a completely inaccurate typing due to probes binding to the novel sequence. Other studies have also shown the ineffectiveness of RSSO in confirming NGS novelties (Smith et al., 2019). These data support that the only current reliable method of confirming novel alleles is on another sequence-based platform. To this end, all novel alleles detected at our site were confirmed by an external ASHI-accredited HLA laboratory using a different commercial kit and NGS platform to avoid assay-related biases. All novelties were in complete concordance between the two laboratories, supporting the use of two independent NGS-based technologies to add validity to the detection of novel sequences.

The accuracy of novel sequences is highly dependent on sequencing quality as well as knowledge of the patient’s disease and specimen type. For example, we observed the presence of a false positive novel allele in two cancer patients due to sequencing DNA extracted from peripheral blood containing leukemic cells. Considering this risk, IPD-IMGT/HLA and National Marrow Donor Program (NMDP) require confirmation of germline DNA for any sequences identified in an individual with hematological malignancy (IPD-IMGT/HLA Database; Be The Match). Inaccurate novel mutations may also be identified due to poor sequencing quality flagged by QC metrics. Indicators of inaccurate novelty identification include poor read coverage, high mismatch count, and multiple novel mutations identified in one sample. In this study, all novel alleles passed relevant QC metrics and only one exonic novelty was observed in each patient. Given the common occurrence of novel alleles and numerous potential assay-related, bioinformatics, and interpretation pitfalls in detection and reporting, the HLA community might consider establishing a formal Proficiency Testing (PT) program to standardize and ensure accuracy in the identification and reporting of novel sequences by accredited laboratories. For example, DNA samples with confirmed novelties using the most updated IPD-IMGT/HLA database can be circulated to accredited laboratories that perform routine NGS typing. In addition to sequence identification, the PT program may further evaluate the laboratory’s ability to describe the mutation (ex. missense, silent, or nonsense point mutations, insertions) and provide a standardized assessment of its effect on the overall protein.

The location and type of a novel mutation are important in determining their effect(s) on the translated HLA protein. In our study, 28% of novelties occurred in key exons and all were single point mutations, which is consistent with other studies and confirms the heterogeneity of these regions (Robinson et al., 2020). The key exons encode the antigen-binding site, which is arguably the most vital genetic region as it directly affects the functional portion of the HLA protein. In addition, the key exons express many of the epitopes bound by HLA antibodies (Duquesnoy, 2006). Less clear are mutations occurring outside the key exons. For example, many novelties were located in exon 1, which encodes the leader peptide. The leader peptide flags newly synthesized HLA proteins to the cell surface and a mutation in exon 1 may affect the protein’s successful translocation (Wu et al., 2020). However, this hypothesis would require experimental cell expression studies to draw any actionable conclusions and the extent to which a clinical HLA laboratory is responsible for this is not defined. Furthermore, this study only included exon novelties and intron variants may have additional implications for protein expression (Alexander et al., 2010). Currently, our center does not routinely report intronic novelties because their clinical relevance in transplantation remains unclear. However, it is known that certain intronic SNPs which affect splicing can greatly alter protein expression. Indeed, the common DRB4*01:03:01:02N allele null variant is caused by an SNP in intron 1 affecting splicing and thus protein expression (Sutton and Knowles, 1990). As the HLA community moves towards sequencing and cataloguing intronic sequences, it may be beneficial to have a standardized approach to the evaluation of intronic SNPs on RNA splicing to improve the understanding of their clinical significance.

In contrast to mutations that lead to null protein expression (i.e., DPA1*01:137N in this study), the clinical significance of missense and silent mutations are less clear. The majority of novelties were missense mutations resulting in an amino acid change. Studies have found that differences in physiochemical structures were associated with adverse outcomes in a transplant rejection setting (Kosmoliaptsis et al., 2016; Wiebe et al., 2018). To investigate the potential effects of this change, the basic properties as defined by the polarity and charge of the original and altered amino acids were compared. Using this method, we observed that four novelties resulted in potentially significant changes to the protein due to a change in the basic properties of the amino acid occurring in an antigen-binding site, which also resulted in an eplet change that may affect antibody binding. The remaining missense novelties did not affect an eplet, however, they included mutations in key exons that resulted in a change of amino acid of different physiochemical properties (one novel allele). Some novelties were presumed to not affect the protein significantly due to a substitution of a similar residue in a non-key exon (seven novel alleles). This approach may serve as an initial strategy to assess the potential clinical significance of amino acid substitution caused by a missense mutation, but will require confirmation by experimental data and correlation with clinical outcomes.

Results of the pan-Canadian survey also highlight the lack of consensus on how to report and communicate novel alleles to the clinical team. Reporting the most related sequence, calling G/P groups, creating a custom allele designation, or reporting the antigen equivalent were methods used for the communication of novel allele results. Furthermore, labs report that the type of mutation and its possible effect on the protein plays a role in how to report. This wide variation in practice demonstrates that there is still a great need from the community for guidance on how to communicate novel alleles to the physician and clinical teams. Future guidelines may standardize a list of minimal assessment criteria (ex. mutation type and location) in the clinical reporting of novel alleles.

The ethnicity of patients with the novel sequences was more diverse than the general Canadian population. This emphasizes the need to encourage and facilitate novel allele submissions by clinical histocompatibility laboratories to ensure IPD-IMGT/HLA continues to maintain a diverse and representative dataset. Indeed, in a recent IPD-IMGT/HLA report, the major contributors to the database were large companies that provide HLA typing services to registries such as for bone marrow donation (Barker et al., 2023). As NGS becomes more widely used in clinical histocompatibility laboratories, this is an invaluable opportunity for local centers to contribute to the community and submit novel sequences that are representative of their population. As highlighted in our survey, there are certain complexities involved when submitting novel sequences. Survey respondents reported a lack of resources and time; the perceived need to re-sequence a second sample; and limitations of gene coverage resulting in complications in reporting as major barriers which hinder their ability to submit novel alleles. Establishing clear requirements on the submission of novel sequences detected specifically by NGS, coupled with improvement and familiarity of tools provided by the NGS analysis software to aid in the creation of required files (ex. FASTA, feature tables), can greatly advance this endeavor.

In this new era of high-throughput NGS technology, the identification of novel sequences is more feasible than ever and will likely accelerate in the future. To ensure that clinical histocompatibility laboratories remain poised to manage this changing landscape, we advocate for developing standards for the verification of novelties, building consensus on the minimum criteria required for clinical evaluation of novel alleles, and updating protocols on the submission of NGS novel sequences to the official database. The HLA community has always adapted to the evolution of laboratory technologies, and we are called upon again to set the standard in the appropriate management of novel sequences in the clinical histocompatibility laboratory.

## Data Availability

The datasets presented in this study can be found in online repositories. The names of the repository/repositories and accession number(s) can be found in the article/Supplementary Material.

## Supplementary material

SUPPLEMENTARY FIGURE S1 The Reverse Sequence-Specific Oligonucleotide (RSSO) reaction pattern for VGH029 that yielded discrepant results from the Next-Generation Sequencing results. The top two panels depict the reaction pattern of the sample followed by the expected reaction patterns for B*56:02:01:01, B*40:01:01, B*40:36, and B*55:08. The red boxes highlight three beads (527, 558, 589) that had an unexpected reaction due to the novel mutation at this position (IMGT codon 116). The novel allele name was B*56:88.
