# Supplementary material for: Novel alleles in the era of next-generation sequencing-based HLA typing calls for standardization and policy
Source: Front Genet. 2023 Oct 13;14:1282834. doi: 10.3389/fgene.2023.1282834 (PMC10611506; doi:10.3389/fgene.2023.1282834)
Supplement: Supplementary file 1 [file Table1.docx]

**Supplemental Table 1.** The basic properties of amino acids used in the analysis of missense novel mutations (Sanvictores and Farci, 2022).

| **Amino acid** | **3 letter abbreviation** | **1 letter abbreviation** | **Basic property** |
| --- | --- | --- | --- |
| Alanine | Ala | A | Nonpolar aliphatic |
| Arginine | Arg | R | Basic |
| Asparagine | Asn | N | Polar uncharged |
| Aspartate | Asp | D | Acidic |
| Cysteine | Cys | C | Polar uncharged |
| Glutamate | Glu | E | Acidic |
| Glutamine | Gln | Q | Polar uncharged |
| Glycine | Gly | G | Nonpolar aliphatic |
| Histidine | His | H | Basic |
| Isoleucine | Ile | I | Nonpolar aliphatic |
| Leucine | Leu | L | Nonpolar aliphatic |
| Lysine | Lys | K | Basic |
| Methionine | Met | M | Nonpolar aliphatic |
| Phenylalanine | Phe | F | Nonpolar aromatic |
| Proline | Pro | P | Polar uncharged |
| Serine | Ser | S | Polar uncharged |
| Threonine | Thr | T | Polar uncharged |
| Tryptophan | Trp | W | Nonpolar aromatic |
| Tyrosine | Tyr | Y | Nonpolar aromatic |
| Valine | Val | V | Nonpolar aliphatic |

**Supplemental Table 2. The list of all samples with a detected novel mutation.** The details of the novel mutations are described here, including the novel allele name, the most related allele, the nucleotide change, IMGT genomic and codon positions, exon position, amino acid change if there was one, and type of mutation. The details regarding the patient or donor the sample with the novel mutation was derived from are also described, including their ethnicity and category. If a novel allele was submitted to IPD-IMGT/HLA, then their GenBank Accession ID is provided. AFA = African American, API = Asian Pacific Islander, CAU = Caucasian, HIS = Hispanic, and NAM = Native American. LIVER = Liver transplant patient, BMD = Bone marrow donor, BMT = Bone marrow transplant patient, DISEASE = Patient tested for genetic disease association of ankylosing spondylitis, KIDNEY = Kidney transplant patient, LD = Living donor for solid organ transplant, DECEASED DONOR = Deceased donor for solid organ transplant.

| **Internal ID** | **Novel Allele Name** | **Included in main cohort** | **Category** | **Most related allele** | **Novel Mutation** | **IMGT Genomic Position** | **IMGT Codon position** | **Exon** | **Amino acid change** | **Mutation** | **Ethnicity** | **GenBank Accession ID** |
| --- | --- | --- | --- | --- | --- | --- | --- | --- | --- | --- | --- | --- |
| **VGH004** | A*03:452 | Yes | LIVER | A*03:05:01 | A>C | 873 | 144 | exon 3 | yes-Lys to Gln | Missense | CAU | BankIt2618150 VGH004 OP352847 |
| **VGH039** | A*26:203 | Yes | BMD | A*26:01:01:01 | G>C | 28 | -15 | exon 1 | yes-Val to Leu | Missense | CAU | Officially named by IPD-IMGT/HLA through submission by other laboratory |
| **VGH035** | B*15:675 | Yes | BMT | B*15:18:01:01 | C>T | 47 | -9 | exon 1 | yes-ala to val | Missense | API | BankIt2715440 VGH035 OR159847 |
| **VGH036** | B*15:675 | Yes | BMD | B*15:18:01:01 | C>T | 47 | -9 | exon 1 | yes-ala to val | Missense | API | BankIt2715440 VGH036 OR159848 |
| **VGH037** | B*15:675 | Yes | BMD | B*15:18:01:01 | C>T | 47 | -9 | exon 1 | yes-ala to val | Missense | API | BankIt2715440 VGH037 OR159849 |
| **VGH005** | B*48:01:12 | Yes | DISEASE | B*48:01:01:01 | C>T | 736 | 97 | exon 3 | no - ser | Silent | NAM | BankIt2618150 VGH005 OP352848 |
| **VGH006** | B*48:01:12 | Yes | LIVER | B*48:01:01:01 | C>T | 736 | 97 | exon 3 | no - ser | Silent | NAM | BankIt2618150 VGH006 OP352849 |
| **VGH002** | B*48:55 | Yes | BMD | B*48:01:01:01 | A>G | 1701 | 228 | exon 4 | yes-Thr to Ala | Missense | NAM | BankIt2618150 VGH002 OP352845 |
| **VGH003** | B*48:55 | Yes | BMD | B*48:01:01:01 | A>G | 1701 | 228 | exon 4 | yes-Thr to Ala | Missense | NAM | BankIt2618150 VGH003 OP352846 |
| **VGH029** | B*56:88 | Yes | KIDNEY | B*56:02:01:01 | A>C | 793 | 116 | exon 3 | yes-leu to phe | Missense | AFA | Officially named by IPD-IMGT/HLA through submission by other laboratory. Sample with discrepant results between RSSO and NGS. |
| **VGH007** | C*05:277 | Yes | BMT | C*05:01:01:02 | G>C | 2721 | 340 | exon 7 | yes-cys to ser | Missense | API | BankIt2618150 VGH007 OP352850 |
| **VGH008** | C*05:277 | Yes | BMD | C*05:01:01:02 | G>C | 2721 | 340 | exon 7 | yes-cys to ser | Missense | API | BankIt2618150 VGH008 OP352851 |
| **VGH009** | C*07:1041 | Yes | BMD | C*07:01:01:01 | C>A | 256 | 17 | exon 2 | yes-arg to ser | Missense | CAU | BankIt2618150 VGH009 OP352852 |
| **VGH001** | C*07:1043 | Yes | KIDNEY | C*07:01:01:01 | T>G | 385 | 60 | exon 2 | yes- Trp to Gly | Missense | CAU | BankIt2613095 VGH001  OP297834 |
| **VGH012** | DPA1*01:03:38:02 | Yes | LIVER | DPA1*01:03:01:04 | C>T | 51 | -15 | exon 1 | no - ala | Silent | CAU | Officially named by IPD-IMGT/HLA through submission by other laboratory |
| **VGH014** | DPA1*01:03:45 | Yes | KIDNEY | DPA1*01:03:01:05 | C>T | 4506 | 169 | exon 3 | no - gly | Silent | CAU | BankIt2620228 VGH014 OP422539 |
| **VGH038** | DPA1*01:106 | Yes | BMD | DPA1*01:03:01:02 | G>A | 5 | -30 | exon 1 | yes- Arg to His | Missense | API | Officially named by IPD-IMGT/HLA through submission by other laboratory |
| **VGH011** | DPA1*01:136 | Yes | BMD | DPA1*01:03:01:02 | A>G | 4463 | 149 | exon 3 | yes-His to Arg | Missense | HIS | BankIt2620228 VGH011 OP422536 |
| **VGH013** | DPA1*01:137N | Yes | LD | DPA1*01:03:01:02 | C>T | 79 | -5 | exon 1 | yes-Arg to STOP | Nonsense | CAU | BankIt2620228 VGH013 OP422538 |
| **VGH040** | DPA1*01:60 | Yes | DECEASED DONOR | DPA1*01:03:01:04 | C>T | 4901 | 224 | exon 4 | yes-arg to Trp | Missense | NAM | Officially named by IPD-IMGT/HLA through submission by other laboratory |
| **VGH010** | DPA1*02:02:13 | Yes | BMD | DPA1*02:02:02:01 | T>C | 4876 | 221 | exon 4 | no - his | Silent | API | BankIt2620228 VGH010 OP422535 |
| **VGH015** | DPA1*02:96 | Yes | KIDNEY | DPA1*02:02:02:01 | A>T | 4850 | 213 | exon 4 | yes-Ile to Phe | Missense | API | BankIt2620228 VGH015 OP422540 |
| **VGH041** | DPB1*1088:01 | Yes | KIDNEY | DPB1*26:01:02 | A>G | 9701 | 194 | exon 4 | yes-Gln to Arg | Missense | API | Officially named by IPD-IMGT/HLA through submission by other laboratory |
| **VGH042** | DQA1*01:01:09:02 | Yes | BMD | DQA1*01:01:01:07 | C>G | 36 | -12 | exon 1 | No - Leu | Silent | API | Officially named by IPD-IMGT/HLA through submission by other laboratory |
| **VGH043** | DQA1*01:01:09:02 | Yes | BMD | DQA1*01:01:01:07 | C>G | 36 | -12 | exon 1 | No - Leu | Silent | API | Officially named by IPD-IMGT/HLA through submission by other laboratory |
| **VGH044** | DQA1*01:01:09:02 | Yes | KIDNEY | DQA1*01:01:01:07 | C>G | 36 | -12 | exon 1 | No - Leu | Silent | API | Officially named by IPD-IMGT/HLA through submission by other laboratory |
| **VGH045** | DQA1*01:01:09:02 | Yes | BMT | DQA1*01:01:01:07 | C>G | 36 | -12 | exon 1 | No - Leu | Silent | API | Officially named by IPD-IMGT/HLA through submission by other laboratory |
| **VGH046** | DQA1*01:01:09:02 | Yes | BMD | DQA1*01:01:01:07 | C>G | 36 | -12 | exon 1 | No - Leu | Silent | API | Officially named by IPD-IMGT/HLA through submission by other laboratory |
| **VGH047** | DQA1*01:01:09:02 | Yes | BMD | DQA1*01:01:01:07 | C>G | 36 | -12 | exon 1 | No - Leu | Silent | API | Officially named by IPD-IMGT/HLA through submission by other laboratory |
| **VGH048** | DQA1*01:01:09:02 | Yes | BMD | DQA1*01:01:01:07 | C>G | 36 | -12 | exon 1 | No - Leu | Silent | API | Officially named by IPD-IMGT/HLA through submission by other laboratory |
| **VGH016** | DQA1*01:02:15 | Yes | DECEASED DONOR | DQA1*01:02:01:11 | C>T | 48 | -8 | exon 1 | no- Thr | Silent | CAU | BankIt2620228 VGH016 OP422541 |
| **VGH020** | DQA1*01:02:16 | Yes | BMD | DQA1*01:02:01:05 | C>A | 3976 | 47 | exon 2 | no - Arg | Silent | API | BankIt2620228 VGH020 OP422545 |
| **VGH019** | DQA1*01:04:08 | Yes | LIVER | DQA1*01:04:01:02 | C>T | 4548 | 99 | exon 3 | no - Pro | Silent | CAU | BankIt2620228 VGH019 OP422544 |
| **VGH022** | DQA1*02:01:15Q | Yes | DECEASED DONOR | DQA1*02:01:01:01 | C>T | 3809 | 14 | exon 2 | no - Asn | Silent | CAU | BankIt2620228 VGH022 OP422547 |
| **VGH021** | DQA1*04:01:07 | Yes | DECEASED DONOR | DQA1*04:01:01:05 | C>T | 48 | -8 | exon 1 | no - thr | Silent | CAU | BankIt2620228 VGH021 OP422546 |
| **VGH017** | DQA1*05:05:16 | Yes | KIDNEY | DQA1*05:05:01:01 | T>A | 69 | -1 | exon 1 | No - gly | Silent | API | BankIt2620228 VGH017 OP422542 |
| **VGH018** | DQA1*05:05:16 | Yes | LD | DQA1*05:05 | T>A | 69 | -1 | exon 1 | No - gly | Silent | API | BankIt2620228 VGH018 OP422543 |
| **VGH023** | DRB1*14:249 | Yes | LD | DRB1*14:04:01:02 | C>T | 8091 | 6 | exon 2 | yes-Arg to Cys | Missense | API | BankIt2620228 VGH023 OP422548 |
| **VGH024** | DRB1*14:54:12 | Yes | DECEASED DONOR | DRB1*14:54:01:03 | A>G | 10752 | 145 | exon 3 | No - thr | Silent | CAU | Officially named by IPD-IMGT/HLA through submission by other laboratory |
| **VGH027** | DRB5*01:130 | Yes | BMD | DRB5*01:02:01 | G>T | 10808 | 160 | exon 3 | yes-Met to Ile | Missense | API | BankIt2620228 VGH027 OP422552 |
| **VGH026** | DRB5*02:37 | Yes | DECEASED DONOR | DRB5*02:02 | A>G | 10732 | 150 | exon 3 | yes - Asn to Ser | Missense | CAU | BankIt2620228 VGH026 OP422551 |
| **VGH049** | A*NOVEL | No | BMT | A*02:06:01:01 | G>T | #N/A | #N/A | exon 3 | yes-gly to val | Missense | N/A | Not submitted to IPD-IMGT/HLA due to novel mutation occurring in leukemic peripheral blood cells and was absent in buccal cells. |
| **VGH050** | B*NOVEL | No | BMT | B*15:01:01:01 | Guanine insertion | #N/A | #N/A | exon 2 | yes-frameshift | Frameshift | N/A | Not submitted to IPD-IMGT/HLA due to novel mutation occurring in leukemic peripheral blood cells and was absent in buccal cells. |
| **VGH025** | DRB3*NOVEL | No | KIDNEY | DRB3*02:02:01:03 | G>A | #N/A | #N/A | exon 2 | no- Arg | Silent | N/A | IMGT was unable to provide an official name because this sample's mutations were silent and it was not fully sequenced. |
