# Supplementary figures and images for: Novel alleles in the era of next-generation sequencing-based HLA typing calls for standardization and policy
Source: Front Genet. 2023 Oct 13;14:1282834. doi: 10.3389/fgene.2023.1282834 (PMC10611506; doi:10.3389/fgene.2023.1282834)

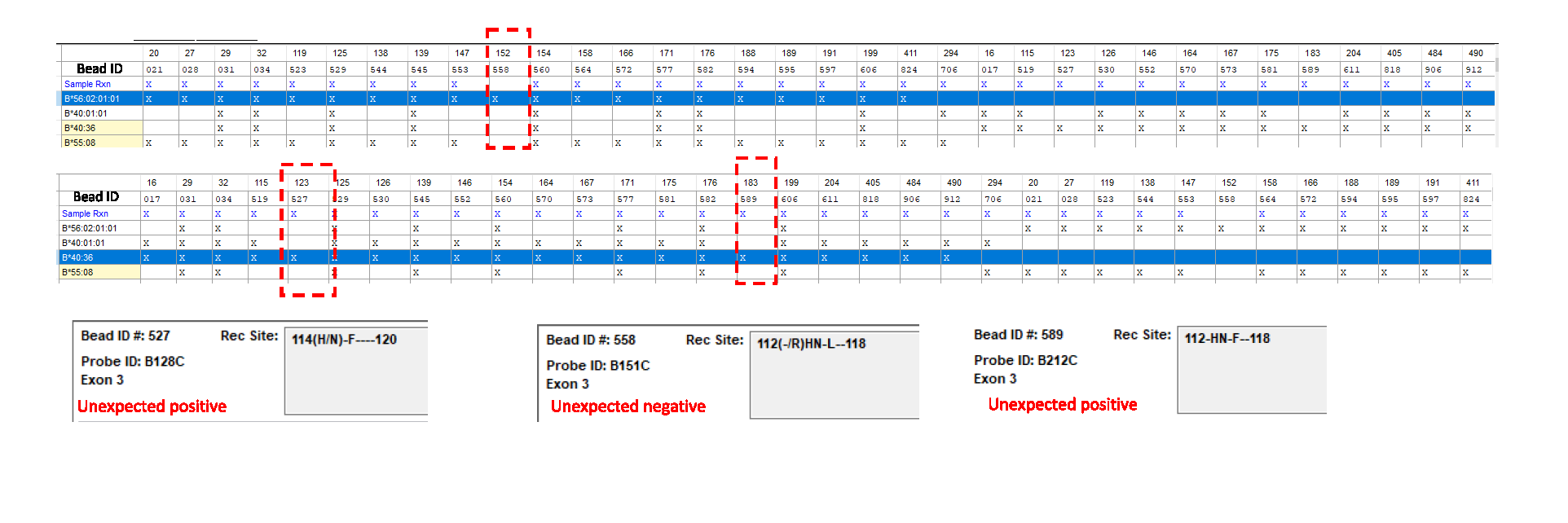

Supplement: Supplementary file 2 [file Image1.TIF]
